# Synthesis, crystal structure and Hirshfeld surface analysis of dimethyl 3-(3-bromo­phen­yl)-6-methyl-7-oxo-3,5,6,7-tetra­hydro­pyrazolo­[1,2-*a*]pyrazole-1,2-di­carboxyl­ate

**DOI:** 10.1107/S2056989021013621

**Published:** 2022-01-07

**Authors:** Rahhal El Ajlaoui, Yassine Hakmaoui, El Mostapha Rakib, El Mostafa Ketatni, Mohamed Saadi, Lahcen El Ammari

**Affiliations:** a Laboratory of Molecular Chemistry, Materials and Catalysis, Faculty of Sciences and Technics, Sultan Moulay Slimane University, Béni-Mellal, BP 523, Morocco; b Département de Chimie, Faculté des Sciences Appliquées Ait Melloul, Université IBN ZOHR, N10. BP 6146 Cité Azrou, Ait Melloul, 86150 Agadir, Morocco; c Higher School of Technology, Sultan Moulay Slimane University, BP 336, Fkih Ben Salah, Morocco; d Laboratoire de Chimie Appliquée des Matériaux, Centre des Sciences des Matériaux, Faculty of Science, Mohammed V University in Rabat, Avenue Ibn Batouta, BP 1014, Rabat, Morocco

**Keywords:** crystal structure, tetra­hydro­pyrazolo­[1,2-*a*]pyrazolone, pyrazole-1,2-di­carboxyl­ate, Hirshfeld surface analysis, hydrogen bonds

## Abstract

The title compound, C_17_H_17_BrN_2_O_5_, resulted from the 1,3-dipolar cyclo­addition reaction between dimethyl acetyl­enedi­carboxyl­ate and (3-bromo­benzyl­idene)-4-methyl-5-oxopyrazolidin-2-ium-1-ide in CHCl_3_. The dihedral angle between the pyrazole rings (all atoms) is 32.91 (10)°; the oxo-pyrazole ring displays an envelope conformation whereas the other pyrazole ring adopts a twisted conformation. The bromo­phenyl ring subtends a dihedral angle of 88.95 (9)° with the mean plane of its attached pyrazole ring. In the crystal, the mol­ecules are linked by C—H⋯O hydrogen bonds and aromatic π–π inter­actions with an inter-centroid distance of 3.8369 (10) Å. The Hirshfeld surface analysis and fingerprint plots reveal that the mol­ecular packing is governed by H⋯H (37.1%), O⋯H/H⋯O (31.3%), Br⋯H/H⋯Br (13.5%) and C⋯H/H⋯C (10.6%) contacts. The energy framework indicates that dispersion energy is the major contributor to the mol­ecular packing.

## Abstract 2

In the title compound, one of the fused pyrazole rings adopts an envelope conformation while the other displays a twisted conformation. In the crystal, the mol­ecules are linked by C—H⋯O hydrogen bonds and aromatic π–π inter­actions.

## Chemical context

Tetra­hydro­pyrazolo­[1,2-*a*] pyrazolo­nes have been studied for about forty years as analogues of penicillin and cephalosporin anti­biotics (Jungheim & Sigmund, 1987 ▸; Jungheim *et al.*, 1987 ▸; Ternansky *et al.*, 1993 ▸; Konaklieva & Plotkin, 2003 ▸; Hanessian *et al.*, 1997 ▸) and have been developed as herbicides and pesticides (Kosower *et al.*, 1995 ▸), as anti­tumor agents and as potent drugs for the treatment of cognitive dysfunctions such as Alzhheimer’s disease. Among a variety of reported synthetic approaches to these compounds (Khidre *et al.*, 2013 ▸; Li & Zhao, 2014 ▸; Svete, 2006 ▸), 1,3-dipolar cyclo­addition has been shown to be effective (Stanley & Sibi, 2008 ▸; Kissane & Maguire, 2010 ▸; Pellissier, 2012 ▸). Until now, several 1,3-dipoles, such as azomethine ylides (El Ajlaoui *et al.*, 2015 ▸), nitro­nes (Jen *et al.*, 2000 ▸; Kano *et al.*, 2005 ▸; Suga *et al.*, 2005 ▸) and carbonyl ylides (Suga *et al.*, 2007 ▸; Nambu *et al.*, 2009 ▸; Padwa 2011 ▸), have been studied. Among them, *N*,*N′*-cyclic azomethine imines (Stanovnik *et al.*, 1998 ▸; Qiu *et al.*, 2014 ▸; Nájera *et al.*, 2015 ▸; Xu & Doyle, 2014 ▸), have been increasingly employed in cyclo­additions for the synthesis of pyrazolo­nes and the related di­nitro­gen-fused heterocyclic derivatives with significant biological activities (Ternansky *et al.*, 1993 ▸; Boyd, 1993 ▸; Muehlebach, *et al.*, 2009 ▸).

As part of our studies in this area, the title compound was synthesized and its mol­ecular and crystal structure and Hirshfeld surface analysis are reported herein. 
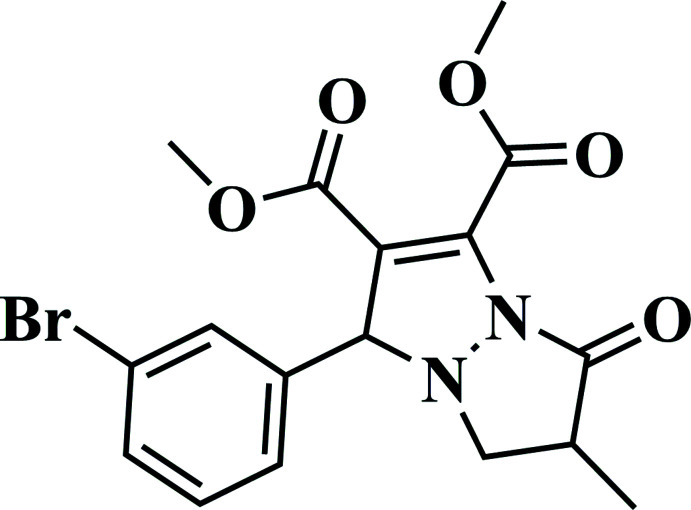


## Structural commentary

**Figure 1:**
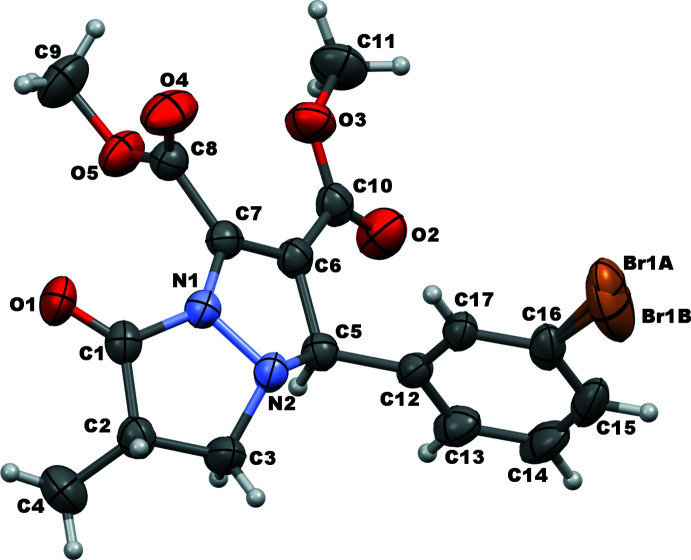
The mol­ecular structure of the title compound showing displacement ellipsoids drawn at the 50% probability level.

The mol­ecular structure of the the title compound is shown in Fig. 1 ▸. There are two stereogenic centres at C2 and C5: in the arbitrarily chosen asymmetric mol­ecule, they have configurations of *S* and *R*, respectively, but a racemic mixture in the crystal is generated in the centrosymmetric *P* 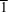 space group. The structure is characterized by a disorder of the Br atom over two adjacent sites [Br⋯Br = 0.32 (2) Å]. The dihedral angle between the fused pyrazole rings (all atoms) is 32.91 (10)°. The C1–C3/N1/N2 oxo-pyrazole ring displays an envelope conformation on C3 whereas the C5–C7/N1/N2 pyrazole ring is twisted on N2—C5, as indicated by the following respective puckering parameters: *Q*(2) = 0.2339 (19) Å, φ(2) = 257.9 (4)° and *Q*(2) = 0.2127 (16) Å, φ(2) = 50.5 (4)°. Moreover, the mean plane passing through the oxo-pyrazole ring subtends a dihedral angle of 61.15 (10)° with the C12–C17 bromo­phenyl ring, which is practically perpendicular to the other pyrazole ring as indicated by the dihedral angle of 88.95 (9)°. The non-H atoms of the ester groups are virtually coplanar, the maximum deviations from the mean planes being 0.017 (2) Å at C10 for the O2/O3/C10/C11 grouping and 0.013 (1) Å at O5 for the O4/O5/C8/C9 grouping. The dihedral angle between these two planes is 62.15 (12)°.

## Supra­molecular features

**Figure 2:**
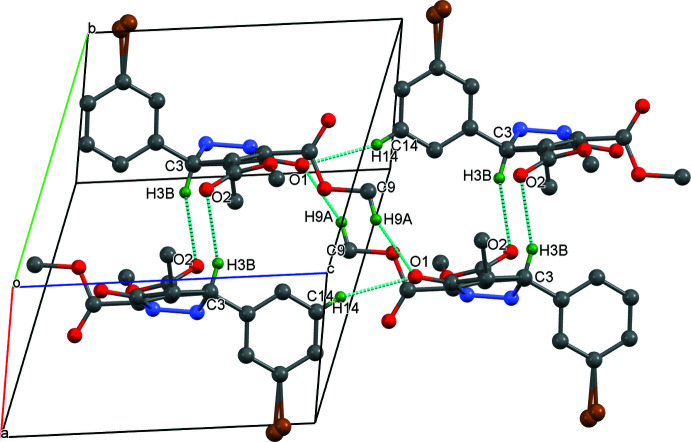
Crystal packing for the title compound showing hydrogen bonds as dashed blue lines.

In the crystal, the mol­ecules are linked by C—H⋯O hydrogen bonds: O1 accepts two such bonds and O2 and O3 accept one each (Table 1 ▸ and Fig. 2 ▸). The bromo­phenyl rings of adjacent mol­ecules are linked by an aromatic stacking π–π inter­action with an inter-centroid distance of 3.8369 (10) Å.

## Database survey

A search of the Cambridge Structural Database (CSD, version 5.42, update of May 2021; Groom *et al.*, 2016 ▸) for the pyrazole-1,2-di­carboxyl­ate unit revealed only one hit, namely refcode RICFUF: dimethyl 3-(*tert*-butyl­amino)-7-phenyl-5-oxo-1*H*,5*H*-pyrazolo­[1,2-*a*]pyrazole-1,2-di­carboxyl­ate (Abbasi *et al.*, 2007 ▸). The conformations of the fused pyrazole rings present in this compound and those of the title compound are different. Furthermore, the values of the dihedral angles between the planes passing through the rings are also very different, except for the angles between the fused pyrazole rings, the difference of which does not exceed one degree, *i.e*. 34.13° in RICFUF and 32.91 (10)° in the title compound. It may be noted that the phenyl substituent is linked to the oxo-pyrazole ring and the two carboxyl­ate groups to the other pyrazole ring in RICFUF, while in the title compound the phenyl and both carboxyl­ate groups are linked to the same pyrazole ring.

## Computational chemistry

**Hirshfeld surface analysis**

**Figure 3:**
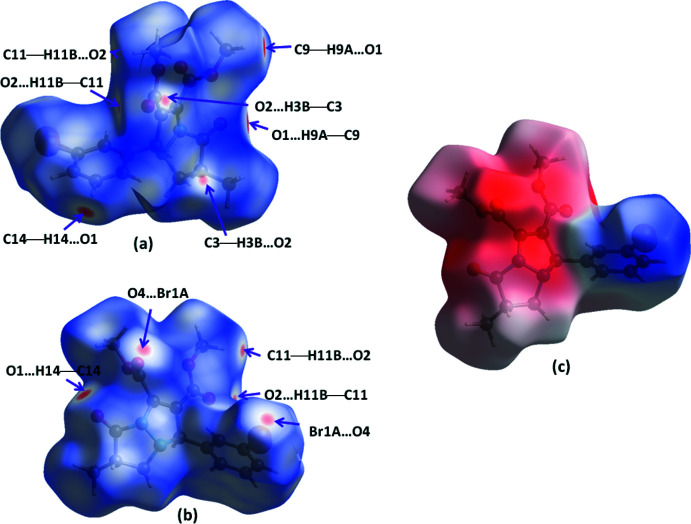
Hirshfeld surfaces of the title compound mapped over (*a*) and (*b*) *d*_norm_ to visualize the inter­molecular C—H⋯O and Br⋯O contacts and (*c*) electrostatic potential energy using the STO-3 G basis set at the Hartree–Fock level.

The Hirshfeld surface (HS) analyses (Spackman & Jayatilaka, 2009 ▸) and two-dimensional fingerprint plots (McKinnon *et al.*, 2007 ▸) generated using *CrystalExplorer17.5* (Turner *et al.*, 2017 ▸) show the various inter­molecular inter­actions in the crystal structure. The three-dimensional *d*_norm_ surface of the title compound using a standard surface resolution with a fixed colour scale of −0.21 to 1.38 a.u is shown in Fig. 3 ▸ *a*,*b*. The intense red spots on the surface are due to the C—H⋯O hydrogen bonds and C—H⋯Br contacts. The bright-red spots in Fig. 3 ▸ *c* indicate atoms with the potential to be hydrogen-bond acceptors (negative electrostatic potential), while blue regions indicate atoms with positive electrostatic potential (hydrogen-bond donors) (Spackman *et al.*, 2008 ▸).

**Figure 4:**
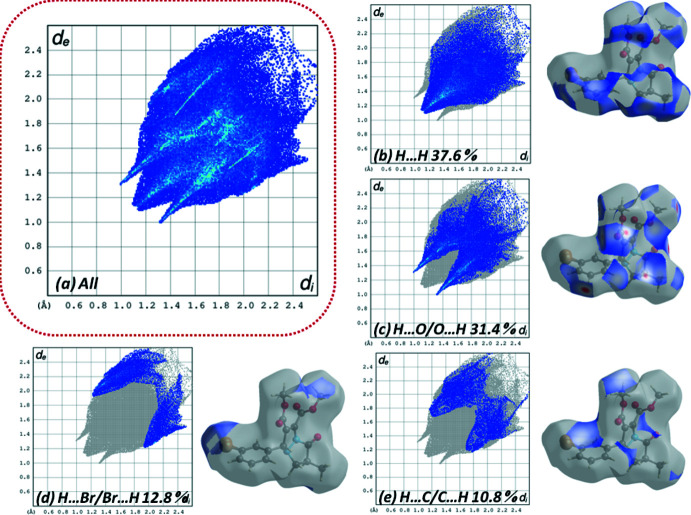
Two-dimensional fingerprint plots for the title compound showing (*a*) all inter­actions, and delineated into (*b*) H⋯H, (*c*) H⋯O/O⋯H, (*d*) H⋯Br/Br⋯H and (*e*) H⋯C/C⋯H inter­actions.

Two-dimensional fingerprint plots for the H⋯H, H⋯O/O⋯H, H⋯Br/Br⋯H and H⋯C/C⋯H contacts are presented in Fig. 4 ▸. The most important inter­action is H⋯H (*d*_e_ = *d*_i_ = 1.15 Å) (Fig. 4 ▸ *b*), contributing 37.6% to the overall crystal packing, which is reflected as widely scattered points of high density due to the large hydrogen content of the mol­ecule. The contribution from the O⋯H/H⋯O contacts (31.4%), corresponding to C—H⋯O inter­actions, is represented by a pair of sharp spikes characteristic of a strong hydrogen-bond inter­action (*d*_i_ + *d*_e_ = 2.40 Å, Fig. 4 ▸ *c*). The reciprocal H⋯Br/Br⋯H inter­actions (12.8%) are present as two symmetrical broad wings with *d*_i_ + *d*_e_ = 3.10 Å (Fig. 4 ▸ *d*). The C⋯H/H⋯C contacts contribute 10.8% to the Hirshfeld surface, featuring a wide region with *d*_i_ + *d*_e_ = 2.95 Å (Fig. 4 ▸ *e*). The smaller percentage contributions of other types of contact are listed in Table 2 ▸.

**Inter­action energy calculations**

**Figure 5:**
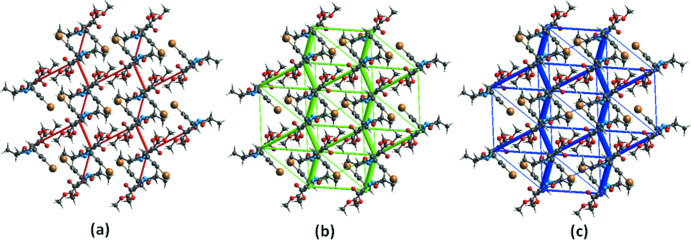
Energy framework of the title compound viewed along [001] showing (*a*) Coulombic energy, (*b*) dispersion energy and (*c*) total energy.

The inter­molecular inter­action energies between mol­ecules in the title compound computed using a B3LYP/6–31G (d, p) energy model available in *Crystal Explorer 17.5* (Turner *et al.*, 2017 ▸), where a cluster of mol­ecules was generated within a radius of 3.8 Å by default. The total inter­molecular energy (*E*_tot_) is the sum of electrostatic (*E*_ele_), polarization (*E*_pol_), dispersion (*E*_dis_), and exchange-repulsion (*E*_rep_) energies. The energy frameworks, which provide a view of the supra­molecular architecture of crystals, are represented by cylinders joining the centroids of mol­ecular pairs using red, green and, blue colour codes for the *E*_ele_, *E*_dis_, and *E*_tot_ energy components, respectively, with a cut-off value of 5 kJ mol^−1^ and a scale factor of 80 to all energy components (Fig. 5 ▸). The benchmarked energies *E*_ele_, *E*_pol_, *E*_dis_ and *E*_rep_ were scaled as 1.057, 0.740, 0.871 and 0.618, respectively (Mackenzie *et al.*, 2017 ▸). The nature and strength of the energies for the key identified inter­molecular inter­actions are summarized in Table 3 ▸. The computed inter­action energies for electrostatic, polarization, dispersion and exchange repulsion are −107.7 kJ mol^−1^, −33.9 kJ mol^−1^, −299.7 kJ mol^−1^ and 185.2 kJ mol^−1^, respectively. These data reveal that the dispersive component makes the major contribution to the inter­molecular inter­actions in the crystal. The calculations showed that the C3—H3*B*⋯O2 hydrogen bond has the greatest energy among all close contacts present in the crystal with its energy (–52.1 kJ mol^−1^) having a major electrostatic contribution (–21.9 kJ mol^−1^). The next most significant contribution, with a total energy of −34.9 kJ mol^−1^, arises from the C11—H11*B*⋯O2 hydrogen bond. Lower energies, compared to the above inter­actions, are calculated for the Br1*A*⋯O4, C9—H9*A*⋯O1 and C14—H14⋯O1 contacts.

**Frontier mol­ecular orbital (FMO) calculations**

The optimized structure of the title compound was established in the gas phase using density functional theory (DFT) using the B3LYP exchange correlation functional and basis-set calculations (Becke, 1993 ▸) as implanted in *GAUSSIAN 09* (Frisch *et al.*, 2009 ▸). The differences between calculated and experimental bond lengths and angles are within a few Ångstroms and degrees, respectively, when compared to the experimental parameters, which indicate that our calculations are acceptable (see supplementary Tables 1 and 2). The HOMO–LUMO gap of the mol­ecule is calculated to be about 4.16 eV.

## Synthesis and crystallization

**Figure 6:**
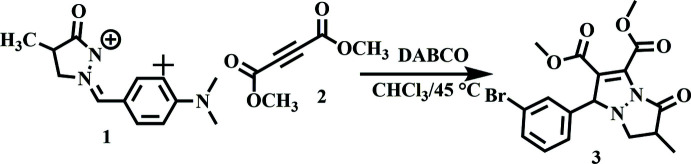
Scheme showing the synthesis of the title compound.

To a solution of DMAD (dimethyl acetyl­enedi­carboxyl­ate; 0.2 mmol, 2 equiv.) in 10 ml of CHCl_3_ containing a catalytic amount of DABCO (0.02 mmol, 0.2 equiv.), (3-bromo­benzyl­idene)-4-methyl-5-oxopyrazolidin-2-ium-1-ide (0.10 mmol, 1 equiv.) was added (Fig. 6 ▸). The mixture was stirred at 318 K until the consumption of the azomethine imine was complete (monitored by TLC with 3:7 hexa­ne/ethyl acetate *v*:*v*). After completion of the reaction, the residue was concentrated *in vacuo*. The crude product was purified by column chromatography on silica gel using hexa­ne:ethyl acetate (2/8 *v*:*v*) as eluent. The title compound was recrystallized from ethanol solution in the form of colourless blocks (yield 68%, m.p. 383 K).

## Refinement

Crystal data, data collection and structure refinement details are summarized in Table 4 ▸. Four reflections affected by the beamstop were omitted from the refinement. All H atoms were placed geometrically (C—H = 0.93–0.98 Å) and refined as riding atoms with *U*_iso_(H) = 1.2*U*_eq_(C) or 1.5*U*_eq_(methyl C). The Br atom was modelled as disordered over adjacent sites in a 0.5862:0.4138 ratio.

## Supplementary Material

Crystal structure: contains datablock(s) I. DOI: 10.1107/S2056989021013621/hb8006sup1.cif

Structure factors: contains datablock(s) I. DOI: 10.1107/S2056989021013621/hb8006Isup2.hkl

Supporting information file. DOI: 10.1107/S2056989021013621/hb8006Isup3.cml

CCDC reference: 2131209

Additional supporting information:  crystallographic information; 3D view; checkCIF report

## Figures and Tables

**Table 1:** Hydrogen-bond geometry (Å, °)

*D*—H⋯*A*	*D*—H	H⋯*A*	*D*⋯*A*	*D*—H⋯*A*
C9—H9*A*⋯O1^i^	0.96	2.42	3.258 (3)	146
C14—H14⋯O1^ii^	0.93	2.53	3.418 (2)	161
C11—H11*B*⋯O2^iii^	0.96	2.60	3.533 (3)	164
C3—H3*B*⋯O2^iv^	0.97	2.62	3.514 (3)	154

Symmetry codes: (i) -x+1, -y+1, -z+2; (ii) x, y, z-1; (iii) -x, -y+1, -z+1; (iv) -x+1, -y+1, -z+1.

**Table 2:** Percentage contributions of inter­atomic contacts to the Hirshfeld surface of the title compound

Contact	Percentage contribution
H⋯H	37.1
O⋯H/H⋯O	31.3
Br⋯H/H⋯Br	13.5
C⋯H/H⋯C	10.6
N⋯H/H⋯N	2.1
O⋯Br/Br⋯O	1.9
C⋯C	1.9
C⋯N/N⋯C	0.7
O⋯N/N⋯O	0.3
Br⋯Br	0.3
N⋯N	0.2

**Table 3:** Summary of inter­action energies (kJ mol^−1^) calculated for the title compound

Contact	*R* (Å)	*E*_ele_	*E*_pot_	*E*_dis_	*E*_rep_	*E*_tot_	Symmetry code
C11—H11*B*⋯O2	7.99	−15.9	−3.6	−36.5	26.5	−34.9	-*x*, −*y*, −*z*
C3—H3*B*⋯O2	6.38	−21.9	−6.9	−54.4	38.0	−52.1	-*x*, −*y*, −*z*
C14—H14⋯O1	11.09	−9.3	−2.6	−11.7	9.8	−15.9	*x*, *y*, *z*
C9—H9*A*⋯O1	11.96	−17.2	−4.4	−16.6	21.4	−22.6	-*x*, −*y*, −*z*
Br1*A*⋯O4	10.81	−14.3	−4.0	−22.4	15.9	−27.7	-*x*, −*y*, −*z*

**Table 4:** Experimental details

Crystal data	
Chemical formula	C_17_H_17_BrN_2_O_5_
*M*_r_	409.23
Crystal system, space group	Triclinic, *P*\overline{1}
Temperature (K)	296
*a*, *b*, *c* (Å)	8.8579 (5), 10.5336 (6), 11.0893 (6)
α, β, γ (°)	62.282 (2), 75.437 (2), 88.241 (2)
*V* (Å^3^)	882.03 (9)
*Z*	2
Radiation type	Mo *K*α
μ (mm^−1^)	2.36
Crystal size (mm)	0.32 × 0.28 × 0.19

Data collection	
Diffractometer	Bruker D8 VENTURE Super DUO
Absorption correction	Multi-scan (*SADABS*; Krause *et al.*, 2015 ▸)
*T*_min_, *T*_max_	0.617, 0.746
No. of measured, independent and observed [*I* > 2σ(*I*)] reflections	27467, 3884, 3257
*R*_int_	0.027
(sin θ/λ)_max_ (Å^−1^)	0.641

Refinement	
*R*[*F* ^2^ > 2σ(*F* ^2^)], *wR*(*F* ^2^), *S*	0.027, 0.078, 1.04
No. of reflections	3882
No. of parameters	235
H-atom treatment	H-atom parameters constrained
Δρ_max_, Δρ_min_ (e Å^−3^)	0.23, −0.26

Computer programs: *APEX3* and *SAINT* (Bruker, 2016 ▸), *SHELXT2014/5* (Sheldrick, 2015*a* ▸), *SHELXL2018/3* (Sheldrick, 2015*b* ▸), *WinGX* and *ORTEP-3 for Windows* (Farrugia, 2012 ▸), *Mercury* (Macrae *et al.*, 2020 ▸) and *publCIF* (Westrip, 2010 ▸).

## supplementary crystallographic information

### Crystal data

**Table:**

C_17_H_17_BrN_2_O_5_	*Z* = 2
*M**_r_* = 409.23	*F*(000) = 416
Triclinic, *P*1	*D*_x_ = 1.541 Mg m^−^^3^
*a* = 8.8579 (5) Å	Mo *K*α radiation, λ = 0.71073 Å
*b* = 10.5336 (6) Å	Cell parameters from 3884 reflections
*c* = 11.0893 (6) Å	θ = 2.2–27.1°
α = 62.282 (2)°	µ = 2.36 mm^−^^1^
β = 75.437 (2)°	*T* = 296 K
γ = 88.241 (2)°	Block, colourless
*V* = 882.03 (9) Å^3^	0.32 × 0.28 × 0.19 mm

### Data collection

**Table:**

Bruker D8 VENTURE Super DUO diffractometer	3884 independent reflections
Radiation source: INCOATEC IµS micro-focus source	3257 reflections with *I* > 2σ(*I*)
HELIOS mirror optics monochromator	*R*_int_ = 0.027
Detector resolution: 10.4167 pixels mm^-1^	θ_max_ = 27.1°, θ_min_ = 2.2°
φ and ω scans	*h* = −11→11
Absorption correction: multi-scan (SADABS; Krause *et al.*, 2015)	*k* = −13→13
*T*_min_ = 0.617, *T*_max_ = 0.746	*l* = −14→14
27467 measured reflections	

### Refinement

**Table:**

Refinement on *F*^2^	Primary atom site location: structure-invariant direct methods
Least-squares matrix: full	Secondary atom site location: difference Fourier map
*R*[*F*^2^ > 2σ(*F*^2^)] = 0.027	Hydrogen site location: inferred from neighbouring sites
*wR*(*F*^2^) = 0.078	H-atom parameters constrained
*S* = 1.04	*w* = 1/[σ^2^(*F*_o_^2^) + (0.0411*P*)^2^ + 0.1854*P*] where *P* = (*F*_o_^2^ + 2*F*_c_^2^)/3
3882 reflections	(Δ/σ)_max_ < 0.001
235 parameters	Δρ_max_ = 0.23 e Å^−^^3^
0 restraints	Δρ_min_ = −0.26 e Å^−^^3^

### Special details

**Table:**

Geometry. All esds (except the esd in the dihedral angle between two l.s. planes) are estimated using the full covariance matrix. The cell esds are taken into account individually in the estimation of esds in distances, angles and torsion angles; correlations between esds in cell parameters are only used when they are defined by crystal symmetry. An approximate (isotropic) treatment of cell esds is used for estimating esds involving l.s. planes.

### Fractional atomic coordinates and isotropic or equivalent isotropic displacement parameters (Å^2^)

**Table:**

	*x*	*y*	*z*	*U*_iso_*/*U*_eq_	Occ. (<1)
C1	0.59723 (18)	0.82071 (16)	0.63241 (16)	0.0400 (3)	
C2	0.73763 (18)	0.89402 (17)	0.50667 (16)	0.0426 (3)	
H2	0.747329	0.997326	0.477490	0.051*	
C3	0.69585 (19)	0.8717 (2)	0.39196 (17)	0.0543 (4)	
H3A	0.734473	0.955697	0.300247	0.065*	
H3B	0.741502	0.788389	0.387941	0.065*	
C4	0.8868 (2)	0.8311 (3)	0.5424 (2)	0.0678 (5)	
H4A	0.974476	0.879267	0.461016	0.102*	
H4B	0.902100	0.844121	0.618805	0.102*	
H4C	0.877991	0.730075	0.570107	0.102*	
C5	0.45427 (17)	0.73166 (15)	0.41577 (15)	0.0372 (3)	
H5	0.533031	0.664725	0.413900	0.045*	
C6	0.32540 (17)	0.65778 (15)	0.55378 (15)	0.0377 (3)	
C7	0.35203 (17)	0.69677 (15)	0.64608 (15)	0.0374 (3)	
C8	0.27063 (18)	0.64773 (17)	0.79956 (16)	0.0418 (3)	
C9	0.2384 (3)	0.4599 (2)	1.03001 (19)	0.0738 (6)	
H9A	0.270856	0.365515	1.078206	0.111*	
H9B	0.273764	0.521748	1.061323	0.111*	
H9C	0.126110	0.453518	1.050550	0.111*	
C10	0.21152 (18)	0.54235 (16)	0.58279 (16)	0.0423 (3)	
C11	−0.0089 (2)	0.3797 (2)	0.7483 (2)	0.0690 (5)	
H11A	−0.084012	0.361710	0.835157	0.103*	
H11B	−0.062337	0.398430	0.677267	0.103*	
H11C	0.047778	0.296859	0.763103	0.103*	
C12	0.40315 (17)	0.79230 (15)	0.28093 (14)	0.0367 (3)	
C13	0.4618 (2)	0.7461 (2)	0.18122 (18)	0.0517 (4)	
H13	0.533604	0.677727	0.196652	0.062*	
C14	0.4127 (3)	0.8027 (2)	0.05800 (19)	0.0644 (6)	
H14	0.453637	0.772789	−0.009399	0.077*	
C15	0.3053 (2)	0.9015 (2)	0.03454 (17)	0.0610 (5)	
H15	0.271768	0.937716	−0.047350	0.073*	
C16	0.2476 (2)	0.94653 (18)	0.13404 (16)	0.0484 (4)	
C17	0.29670 (17)	0.89409 (16)	0.25580 (15)	0.0394 (3)	
H17	0.258100	0.927269	0.320979	0.047*	
N1	0.47794 (14)	0.80099 (13)	0.58181 (12)	0.0385 (3)	
N2	0.52250 (15)	0.84889 (13)	0.43155 (12)	0.0392 (3)	
O1	0.58506 (15)	0.78306 (15)	0.75542 (12)	0.0574 (3)	
O2	0.22293 (17)	0.48679 (14)	0.50806 (14)	0.0631 (4)	
O3	0.09960 (14)	0.50348 (13)	0.70232 (13)	0.0560 (3)	
O4	0.18874 (17)	0.72005 (15)	0.83908 (14)	0.0664 (4)	
O5	0.30539 (15)	0.51832 (13)	0.87923 (11)	0.0537 (3)	
Br1A	0.08949 (17)	1.08438 (13)	0.11124 (14)	0.05769 (15)	0.5862
Br1B	0.1173 (3)	1.0792 (3)	0.0900 (3)	0.1081 (10)	0.4138

### Atomic displacement parameters (Å^2^)

**Table:**

	*U*^11^	*U*^22^	*U*^33^	*U*^12^	*U*^13^	*U*^23^
C1	0.0449 (8)	0.0403 (8)	0.0409 (8)	0.0084 (6)	−0.0182 (6)	−0.0210 (6)
C2	0.0429 (8)	0.0422 (8)	0.0438 (8)	0.0011 (6)	−0.0158 (6)	−0.0188 (7)
C3	0.0417 (8)	0.0765 (12)	0.0409 (8)	−0.0126 (8)	−0.0039 (7)	−0.0272 (8)
C4	0.0453 (10)	0.0867 (15)	0.0733 (13)	0.0103 (9)	−0.0232 (9)	−0.0359 (11)
C5	0.0362 (7)	0.0378 (7)	0.0373 (7)	0.0047 (5)	−0.0116 (6)	−0.0168 (6)
C6	0.0399 (7)	0.0346 (7)	0.0349 (7)	0.0042 (6)	−0.0137 (6)	−0.0117 (6)
C7	0.0356 (7)	0.0366 (7)	0.0359 (7)	0.0069 (5)	−0.0108 (6)	−0.0134 (6)
C8	0.0396 (8)	0.0439 (8)	0.0382 (8)	0.0050 (6)	−0.0104 (6)	−0.0167 (7)
C9	0.0947 (16)	0.0698 (13)	0.0363 (9)	0.0047 (11)	−0.0136 (9)	−0.0103 (9)
C10	0.0438 (8)	0.0374 (7)	0.0412 (8)	0.0031 (6)	−0.0168 (7)	−0.0121 (6)
C11	0.0573 (11)	0.0614 (12)	0.0733 (13)	−0.0196 (9)	−0.0058 (10)	−0.0241 (10)
C12	0.0371 (7)	0.0409 (7)	0.0307 (7)	−0.0047 (6)	−0.0057 (6)	−0.0171 (6)
C13	0.0526 (10)	0.0586 (10)	0.0471 (9)	−0.0028 (8)	−0.0030 (7)	−0.0322 (8)
C14	0.0738 (13)	0.0832 (14)	0.0400 (9)	−0.0215 (11)	0.0041 (9)	−0.0400 (10)
C15	0.0740 (13)	0.0699 (12)	0.0313 (8)	−0.0236 (10)	−0.0151 (8)	−0.0154 (8)
C16	0.0523 (9)	0.0460 (8)	0.0394 (8)	−0.0105 (7)	−0.0199 (7)	−0.0095 (7)
C17	0.0414 (8)	0.0450 (8)	0.0320 (7)	−0.0012 (6)	−0.0116 (6)	−0.0173 (6)
N1	0.0387 (6)	0.0437 (7)	0.0328 (6)	0.0016 (5)	−0.0103 (5)	−0.0173 (5)
N2	0.0428 (7)	0.0424 (6)	0.0310 (6)	−0.0011 (5)	−0.0130 (5)	−0.0144 (5)
O1	0.0644 (8)	0.0741 (8)	0.0392 (6)	0.0039 (6)	−0.0209 (5)	−0.0274 (6)
O2	0.0767 (9)	0.0566 (7)	0.0574 (7)	−0.0117 (6)	−0.0126 (6)	−0.0297 (6)
O3	0.0482 (7)	0.0557 (7)	0.0564 (7)	−0.0121 (5)	−0.0033 (6)	−0.0245 (6)
O4	0.0697 (9)	0.0660 (8)	0.0554 (7)	0.0218 (7)	−0.0032 (6)	−0.0297 (7)
O5	0.0660 (8)	0.0486 (6)	0.0363 (6)	0.0124 (5)	−0.0126 (5)	−0.0128 (5)
Br1A	0.0563 (3)	0.0541 (3)	0.0611 (2)	0.0139 (2)	−0.0361 (2)	−0.0164 (2)
Br1B	0.1041 (15)	0.0955 (10)	0.1272 (17)	0.0249 (8)	−0.0824 (13)	−0.0302 (9)

### Geometric parameters (Å, º)

**Table:**

C1—O1	1.2062 (18)	C9—H9A	0.9600
C1—N1	1.3765 (19)	C9—H9B	0.9600
C1—C2	1.507 (2)	C9—H9C	0.9600
C2—C4	1.519 (2)	C10—O2	1.202 (2)
C2—C3	1.527 (2)	C10—O3	1.329 (2)
C2—H2	0.9800	C11—O3	1.447 (2)
C3—N2	1.481 (2)	C11—H11A	0.9600
C3—H3A	0.9700	C11—H11B	0.9600
C3—H3B	0.9700	C11—H11C	0.9600
C4—H4A	0.9600	C12—C17	1.381 (2)
C4—H4B	0.9600	C12—C13	1.387 (2)
C4—H4C	0.9600	C13—C14	1.392 (3)
C5—N2	1.4903 (19)	C13—H13	0.9300
C5—C12	1.5110 (19)	C14—C15	1.367 (3)
C5—C6	1.523 (2)	C14—H14	0.9300
C5—H5	0.9800	C15—C16	1.375 (3)
C6—C7	1.338 (2)	C15—H15	0.9300
C6—C10	1.466 (2)	C16—C17	1.380 (2)
C7—N1	1.3801 (19)	C16—Br1B	1.754 (3)
C7—C8	1.507 (2)	C16—Br1A	1.961 (2)
C8—O4	1.190 (2)	C17—H17	0.9300
C8—O5	1.3131 (19)	N1—N2	1.4444 (16)
C9—O5	1.447 (2)		
			
O1—C1—N1	124.11 (15)	H9A—C9—H9C	109.5
O1—C1—C2	129.17 (14)	H9B—C9—H9C	109.5
N1—C1—C2	106.72 (12)	O2—C10—O3	124.51 (15)
C1—C2—C4	110.91 (14)	O2—C10—C6	122.90 (15)
C1—C2—C3	103.61 (12)	O3—C10—C6	112.54 (14)
C4—C2—C3	114.19 (15)	O3—C11—H11A	109.5
C1—C2—H2	109.3	O3—C11—H11B	109.5
C4—C2—H2	109.3	H11A—C11—H11B	109.5
C3—C2—H2	109.3	O3—C11—H11C	109.5
N2—C3—C2	106.03 (12)	H11A—C11—H11C	109.5
N2—C3—H3A	110.5	H11B—C11—H11C	109.5
C2—C3—H3A	110.5	C17—C12—C13	119.18 (14)
N2—C3—H3B	110.5	C17—C12—C5	120.30 (12)
C2—C3—H3B	110.5	C13—C12—C5	120.52 (15)
H3A—C3—H3B	108.7	C12—C13—C14	119.69 (18)
C2—C4—H4A	109.5	C12—C13—H13	120.2
C2—C4—H4B	109.5	C14—C13—H13	120.2
H4A—C4—H4B	109.5	C15—C14—C13	121.00 (16)
C2—C4—H4C	109.5	C15—C14—H14	119.5
H4A—C4—H4C	109.5	C13—C14—H14	119.5
H4B—C4—H4C	109.5	C14—C15—C16	118.90 (16)
N2—C5—C12	110.97 (11)	C14—C15—H15	120.5
N2—C5—C6	101.25 (11)	C16—C15—H15	120.5
C12—C5—C6	116.58 (12)	C15—C16—C17	121.17 (17)
N2—C5—H5	109.2	C15—C16—Br1B	115.19 (16)
C12—C5—H5	109.2	C17—C16—Br1B	123.57 (16)
C6—C5—H5	109.2	C15—C16—Br1A	121.65 (14)
C7—C6—C10	127.27 (14)	C17—C16—Br1A	117.17 (13)
C7—C6—C5	109.39 (13)	Br1B—C16—Br1A	7.44 (12)
C10—C6—C5	122.35 (13)	C16—C17—C12	120.03 (14)
C6—C7—N1	110.20 (12)	C16—C17—H17	120.0
C6—C7—C8	131.71 (14)	C12—C17—H17	120.0
N1—C7—C8	118.09 (13)	C1—N1—C7	130.55 (13)
O4—C8—O5	126.49 (15)	C1—N1—N2	114.06 (11)
O4—C8—C7	123.05 (14)	C7—N1—N2	109.22 (11)
O5—C8—C7	110.44 (13)	N1—N2—C3	103.78 (11)
O5—C9—H9A	109.5	N1—N2—C5	105.01 (10)
O5—C9—H9B	109.5	C3—N2—C5	116.32 (13)
H9A—C9—H9B	109.5	C10—O3—C11	116.75 (14)
O5—C9—H9C	109.5	C8—O5—C9	116.16 (15)

### Hydrogen-bond geometry (Å, º)

**Table:**

*D*—H···*A*	*D*—H	H···*A*	*D*···*A*	*D*—H···*A*
C9—H9*A*···O1^i^	0.96	2.42	3.258 (3)	146
C14—H14···O1^ii^	0.93	2.53	3.418 (2)	161
C11—H11*B*···O2^iii^	0.96	2.60	3.533 (3)	164
C3—H3*B*···O2^iv^	0.97	2.62	3.514 (3)	154

Symmetry codes: (i) −*x*+1, −*y*+1, −*z*+2; (ii) *x*, *y*, *z*−1; (iii) −*x*, −*y*+1, −*z*+1; (iv) −*x*+1, −*y*+1, −*z*+1.

### Comparison of the selected experimental and calculated geometric parameters (Å, °)

**Table:**

Bond lengths	*X-ray*	*DFT*
		
O1—C1	1.206 (2)	1.209
C1—N1	1.377 (2)	1.384
N1—N2	1.444 (2)	1.438
N1—C7	1.380 (2)	1.374
C3—N2	1.481 (2)	1.470
C5—N2	1.490 (2)	1.484
C8—O4	1.190 (2)	1.199
C8—O5	1.313 (2)	1.334
C10—O2	1.202 (2)	1.212
C10—O3	1.329 (2)	1.351
O3—C11	1.447 (2)	1.438
O5—C9	1.447 (2)	1.443
C16—Br1A/Br1B	1.961 (2)/1.754 (3)	1.922
Angles		
O1—C1—N1	124.1 (2)	125.7
O1—C1—C2	129.2 (2)	129.2
N1—C1—C2	106.7 (2)	105.1
C1—N1—C7	130.6 (2)	133.8
C1—N1—N2	114.1 (2)	113.1
C3—N2—C5	116.3 (2)	119.8
C7—C8—O4	123.1 (2)	122.9
C7—C8—O5	110.4 (2)	110.8
O4—C8—O5	126.5 (2)	126.3
C6—C10—O2	122.9 (2)	123.9
C6—C10—O3	112.5 (2)	112.4
O2—C10—O3	124.5 (2)	123.6
C10—O3—C11	116.8 (2)	115.7
C8—O5—C9	116.2 (2)	115.2
C15—C16—Br1A/Br1B	121.7 (2)/115.2 (2)	119.2
C17—C16—Br1A/Br1B	117.2 (2)/123.6 (2)	119.1

### Quantum chemical parameters of the title compound calculated by B3LYP/6-311G (d, p)

**Table:**

E_T_(eV)	101131.745
E_HOMO_ (eV)	–6.026
E_LUMO_ (eV)	–1.868
DE_(LUMO–HOMO)_ (eV)	4.158
Chemical hardness (η)	2.079
Chemical Softness (ξ)	0.241
Chemical potential (µ)	3.947
Electrophilicity (ψ)	3.754
Electronegativity (χ)	–3.947
Dipole moment (D)	3.847

η = 1/2[E_LUMO_-E_HOMO_], ξ = 1/2η, µ = [1/2(E_LUMO_+E_HOMO_)], ψ = µ^2^/2η, χ = –µ
